# Bacterial Pathogens Activate a Common Inflammatory Pathway through IFNλ Regulation of PDCD4

**DOI:** 10.1371/journal.ppat.1003682

**Published:** 2013-10-03

**Authors:** Taylor S. Cohen, Alice S. Prince

**Affiliations:** Department of Pediatrics, Columbia University, New York, New York, United States of America; Geisel School of Medicine at Dartmouth, United States of America

## Abstract

The type III interferon (IFNλ) receptor IL-28R is abundantly expressed in the respiratory tract and has been shown essential for host defense against some viral pathogens, however no data are available concerning its role in the innate immune response to bacterial pathogens. *Staphylococcus aureus* and *Pseudomonas aeruginosa* induced significant production of IFNλ in the lung, and clearance of these bacteria from the lung was significantly increased in IL-28R null mice compared to controls. Improved bacterial clearance correlated with reduced lung pathology and a reduced ratio of pro- vs anti-inflammatory cytokines in the airway. In human epithelial cells IFNλ inhibited miR-21 via STAT3 resulting in upregulation of PDCD4, a protein known to promote inflammatory signaling. In vivo 18 hours following infection with either pathogen, miR-21 was significantly reduced and PDCD4 increased in the lungs of wild type compared to IL-28R null mice. Infection of PDCD4 null mice with USA300 resulted in improved clearance, reduced pathology, and reduced inflammatory cytokine production. These data suggest that during bacterial pneumonia IFNλ promotes inflammation by inhibiting miR-21 regulation of PDCD4.

## Author Summary

The role of interferons (types I, II, and III) in viral and bacterial infections has been a topic of intense research over the last decade. The contribution of the type I interferons during bacterial pneumonias particularly has been shown to be highly variable depending on the specific pathogen. Our data for the first time demonstrate that type III interferon plays a significant role in the pathogenesis of bacterial pneumonia, and its contribution is similar in both Gram positive and Gram negative infections. We show in epithelial cells that miR-21 and PDCD4 are downstream effectors of type III interferon that prolong production of inflammatory cytokines. Utilizing mice that lack the receptor for type III interferon or PDCD4, we show that inhibiting this pathway improves bacterial clearance from the airways and lung tissue. These data suggest novel targets for therapy in a variety of bacterial pneumonias.

## Introduction

The interferon (IFN) family is composed of three subgroups (types I, II, and III IFN), and through their distinct receptors, IFNs signal through STAT transcription factors to upregulate expression of over 300 IFN dependent genes. In the lung bacterial pathogen associated molecular patterns (PAMPs) can be internalized and thus gain access to the intracellular receptors involved in type I IFN signaling [1]–[3]. Activation of type I IFN signaling can be either protective or detrimental to the host depending on the specific pathogen [4]–[9]. Type I IFNs promote the pathogenesis of *Staphylococcus aureus* pulmonary infection through upregulation of CXCR3 chemokines and T-cell recruitment while improving eradication of *Pseudomonas aeruginosa* by reducing inflammasome signaling [1], [10]–[16].

Interferons induce expression of downstream genes through distinct receptors. Type I IFN signals through the ubiquitously expressed interferon-α/β receptor (IFNAR), while the type III IFN (IFNλ) family, composed of IL-28A/B and IL-29, signals through the more cell specific receptor complex of IL-10R2 and IL-28R [17]–[21]. Following activation of either IFN pathway, an autocrine signaling network mediates the cellular response, primarily through JAK/STAT signaling and the induction of IFN dependent gene expression. It would seem that the two IFNs activate redundant downstream signaling pathways. However altered signaling kinetics and the limited distribution of IL-28R, restricted primarily to mucosal tissues, suggest distinct roles for type I and type III IFN depending on the infection site [22]–[28]. For example in the lung type III IFN is the primary IFN produced by respiratory epithelial cells in response to viral stimulation and is required for clearance of influenza from the airway [29]–[32]. Several of the immunological effects of IFNλ such as upregulation of MHC I and II, induction of NF-κB dependent cytokine production and effects on DC maturation and differentiation, could be highly relevant to the pathogenesis of bacterial infection [33]–[36].

Interferon signaling is linked to the regulation of micro RNAs, small non-coding RNA inhibitors of mRNA translation capable of directly influencing innate immune signaling [4]–[9]. In tumor cells, miR-21 targets the tumor suppressor programmed cell death protein 4 (PDCD4) promoting tumor growth and contributing to the inflammatory tumor microenvironment [1], [10]–[16]. PDCD4 represses translation of cellular mRNA through its binding to eukaryotic initiation factor 4A (eIF4A) [17]–[21]. Phosphorylation of PDCD4 by the ribosomal S6 kinase (p70S6K) results in release of eIF4A from PDCD4, ubiquitin dependent degredation of PDCD4, and enhanced mRNA translation [22]–[28]. Inflammatory cytokine production in response to toll like receptor (TLR) 2 or 4 activation by decorin or lipopolysaccharide (LPS) is significantly influenced by expression of miR-21 and PDCD4 [29]–[32]. Therefore the pro-inflammatory contribution of PDCD4 to host signaling during bacterial pneumonia could significantly contribute to lung pathology [33]–[36].

In the experiments detailed in this report, we examined the importance of type III IFNs in the innate immune response to two major airway pathogens, USA300 MRSA and *P. aeruginosa*, currently the most common causes of ventilator associated pneumonia [37], [38]. We found that IFNλ is induced in the course of bacterial airway infection and results in downregulation of the microRNA miR-21, sustained expression of PDCD4, and increased proinflammatory cytokine production. Mice lacking the IFNλ receptor, IL-28R, or PDCD4 had significantly improved clearance of both airway pathogens and less pulmonary pathology associated with reduced levels of inflammatory cytokines.

## Results

### IL-28R^−/−^ mice are more resistant to *S. aureus* respiratory infection

**Figure 1:**
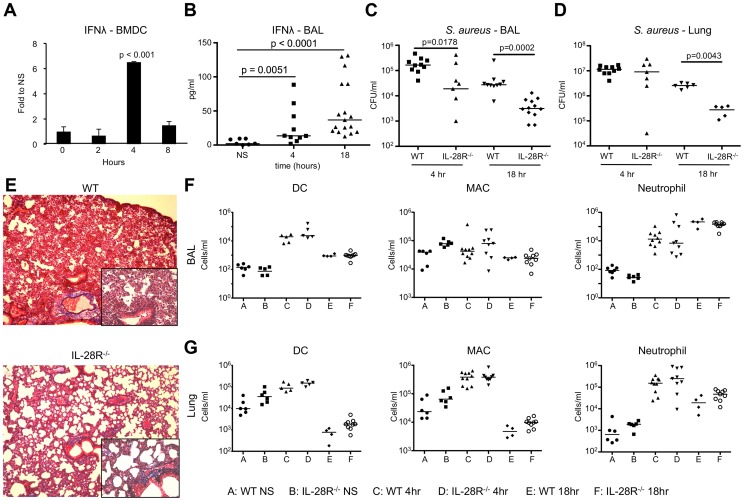
Induction of IFNλ by *S. aureus* USA300 inhibits bacterial clearance. (A) qRT-PCR analysis of IFNλ mRNA in BMDCs stimulated with heat killed USA300, µ ± SD. (B) ELISA analysis of IFNλ in BAL of WT mice following 4 and 18 hours of infection with USA300. (C) Numbers (CFU) of USA300 recovered from BAL of WT and IL-28R^−/−^ mice following 4 and 18 hours of infection. (D) Numbers (CFU) of USA300 recovered from lung tissue of WT and IL-28R^−/−^ mice following 4 and 18 hours of infection. (E) Trichrome stained lungs from WT and IL-28R^−/−^ mice following and 18 hour USA300 infection (original magnification 100x, insert 400x). (F) Numbers of dendritic cells (DC), macrophages (MAC), and neutrophils in BAL of WT and IL-28R^−/−^ in unstimulated (NS) mice or following 4 or 18 hours of infection. (G) Numbers of dendritic cells (DC), macrophages (MAC), and neutrophils in lung tissue of WT and IL-28R^−/−^ in unstimulated (NS) mice or following 4 or 18 hours of infection. Data are representative of at least 2 independent experiments.

In vitro studies were performed to establish the kinetics of IFNλ induction in response to the extracellular bacterial pathogen *S. aureus*. A significant increase in IFNλ transcript (p < 0.001) was observed in wild type (WT) bone marrow derived dendritic cells (BMDCs) following 4 hours of stimulation with heat killed USA300, which returned to baseline by 8 hours (**Fig. 1A**). Similar induction was observed when BMDCs were stimulated with live bacteria (MOI 100, 4 hours), and induction was significantly reduced (p = 0.0062) in IL-28R^−/−^ BMDCs (**Fig. S1A**).

To evaluate the role of type III IFN in MRSA pneumonia we infected WT C57BL/6 and IL-28R^−/−^ mice intranasally with 1×10^7^ CFU USA300. IFNλ mRNA levels increased significantly by 4 hours in the lung of WT mice (p = 0.0047) (**Fig. S1B**), and protein levels were significantly increased in the airway of WT mice by 4 (p = 0.0051) and 18 (p < 0.0001) hours post infection (**Fig. 1B**). By 4 hours following intranasal infection the numbers of bacteria recovered from the airway were significantly reduced in IL-28R^−/−^ mice compared with control (p  = 0.0178) (**Fig. 1C**), a difference not observed in the lung tissue (**Fig. 1D**). By 18 hours significantly fewer bacteria were recovered from both the airway (p = 0.0002) and tissue (p = 0.0043) of knockout mice compared to control. Lung pathology was reduced in IL-28R^−/−^ compared to WT controls as determined by trichrome stained lung sections (**Fig. 1E**). The numbers of dendritic cells, macrophages, or neutrophils recruited to the airway (**Fig. 1F**) or lung tissue (**Fig. 1G**) were not affected by lack of IL-28R, suggesting that differences in bacterial clearance and pathology were not due to increased numbers of phagocytotic cells.

### Expression of inflammatory cytokines is enhanced by type III IFN

**Figure 2:**
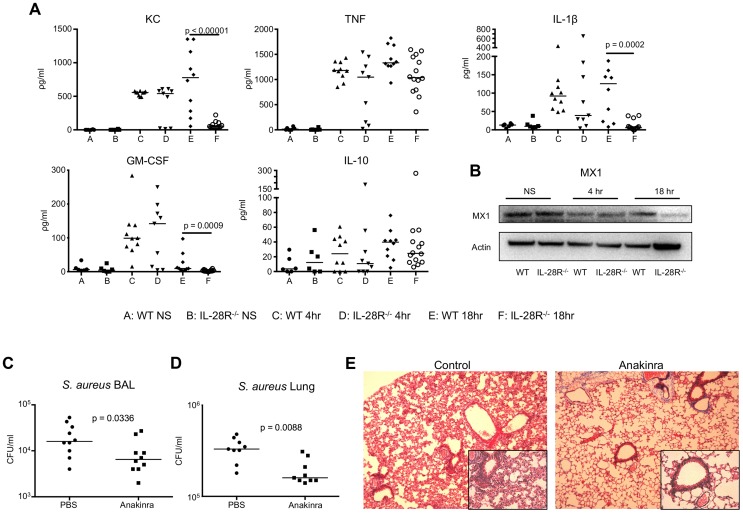
Cytokine production during USA300 infection contributes to pathology. (A) ELISA analysis of individual cytokines in BAL of WT and IL-28R^−/−^ mice. (B) Western blot analysis of MX1 expression in lungs of WT and IL-28R^−/−^ mice following 4 and 18 hours of infection with USA300. (C) Numbers (CFU) of USA300 recovered from the BAL of from PBS control and Anakinra pre-treated mice following an 18 hour infection. (D) Numbers of USA300 (CFU) recovered from the lung of from PBS control and Anakinra pre-treated mice following an 18 hour infection. (E) Trichrome stained lungs from PBS control and Anakinra pre-treated mice following and 18 hour USA300 infection (original magnification 100x, insert 400x). Data are representative of at least 2 independent experiments.

Type III IFN activates a family of over 300 genes through a Jak-Stat signaling cascade [39], [40]. We analyzed expression of cytokines in the bronchial alveolar lavage (BAL) of wild type and IL-28R^−/−^ mice following 4 and 18 hour MRSA infection by ELISA. Significant differences in cytokine expression were not observed following a short (4 hour) infection suggesting that initial activation of cytokine production does not depend on signaling through IL-28R. By 18 hours significant reductions in KC (p < 0.0001), GM-CSF (p = 0.0009) and IL-1β (p = 0.0002) but not TNF or IL-10 were observed in IL-28R^−/−^ as compared with WT mice (**Fig. 2A**). Levels of the interferon response gene MX1 were reduced in IL-28R−/− mice compared to WT at the 18 hour time point, confirming the inhibition of interferon signaling (**Fig. 2B**). Installation of recombinant IFNλ did not significantly increase cytokine expression in the BAL of uninfected mice (**Fig. S2**). Therefore it appears that IFNλ does not directly induce cytokine production, but is more likely involved in the regulation of inflammatory cytokines during acute *S. aureus* infection.

The pro-inflammatory cytokine IL-1β has shown to promote lung injury during *P. aeruginosa* pneumonia, and was significantly decreased in the airways of *S. aureus* infected IL-28R^−/−^ mice [11]. To determine if decreased IL-1β was similarly associated with the improved outcome of the IL-28R^−/−^ mice, we predicted that Anakinra, a synthetic IL-1R antagonist, would improve clearance of *S. aureus* from the lung and reduce lung pathology. Numbers of bacteria recovered 18 hours following infection with USA300 were significantly lower from the airway (p = 0.0336) (**Fig. 2C**) and lung (p = 0.0088) (**Fig. 2D**) of Anakinra treated compared to PBS pretreated controls. Lung pathology was reduced in Anakinra treated mice following an 18 hour infection compared to infected control mice (**Fig. 2E**). These data suggest that the reduced pathology observed in IL-28R null mice was due in part to reduction in the inflammatory cytokine IL-1β.

### Type III IFN regulates PDCD4 expression in epithelial cells

**Figure 3:**
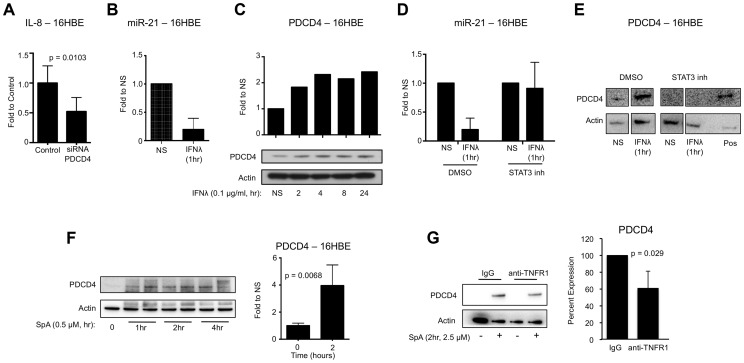
IFNλ regulation of miR-21 and PDCD4. (A) qRT-PCR analysis of IL-8 in 16HBE cells treated with control or PDCD4 siRNA and infected with USA300, µ ± SD. (B) qRT-PCR analysis of miR-21 in 16HBE cells treated with recombinant IFNλ for 1 hour, µ ± SD. (C) Western blot analysis of PDCD4 in 16HBE cells treated with recombinant IFNλ. (D) qRT-PCR analysis of miR-21 in 16HBE cells pretreated with STAT3 inhibitor or DMSO treated with recombinant IFNλ for 1 hour, µ ± SD. (E) Western blot analysis of PDCD4 in 16HBE cells pretreated with STAT3 inhibitor or DMSO treated with recombinant IFNλ for 1 hour. Lysate from 16HBE cells treated with DMSO and IFNλ was used as a positive control (pos). (F) Western blot analysis of PDCD4 in human epithelial cells (16HBE) following stimulation with *S. aureus* protein A (SpA), µ ± SD. (G) Western blot analysis of PDCD4 in 16HBE cells treated with anti-TNFR1 or control IgG and SPA for 2 hours, µ ± SD. Data are representative of at least 2 independent experiments.

PDCD4 has been shown to promote inflammatory cytokine production in response to LPS, and reduction of PDCD4 expression in human epithelial cell monolayers (16HBE) significantly reduced IL-8 induction (p = 0.0103) in response to USA300 (**Fig. 3A**) [31]. Since PDCD4 expression is regulated by the micro RNA miR-21, we tested whether IFNλ promotes inflammatory signaling by inhibiting miR-21 expression. In vitro, miR-21 expression in 16HBEs was reduced following 1 hour treatment with recombinant IFNλ (**Fig. 3B**), which correlated with an increase in PDCD4 protein levels (**Fig. 3C**). Reduction of miR-21 (**Fig. 3D**) and increased PDCD4 expression (**Fig. 3E**) following IFNλ treatment was dependent on STAT3 phosphorylation.

### *S. aureus* SpA induced PDCD4 expression

In response to acute LPS exposure macrophages upregulate PDCD4 expression [31]. *S. aureus*, as a Gram positive pathogen, can induce inflammatory signaling in epithelial cells through interaction of its major surface component protein A (SpA) and host receptor TNFR1 [13], [41]–[43]. We hypothesized that SpA-TNFR1 interaction would upregulate PDCD4 expression and promote production of inflammatory cytokines. 16HBE epithelial cells were exposed to *S. aureus* protein A (SpA) and increases in the expression of PDCD4 (p = 0.0068) were observed (**Fig. 3F**). To confirm the relationship between TNFR1 and PDCD4, we pre-treated 16HBEs with antibody to the extracellular domain of TNFR1 and demonstrated significantly reduced induction of PDCD4 by SpA compared to IgG pretreatment (p = 0.029) (**Fig. 3G**). These data demonstrate that SpA, like LPS, is able to induce expression of PDCD4 in host cells.

### PDCD4 expression inversely correlates with clearance of USA300

**Figure 4:**
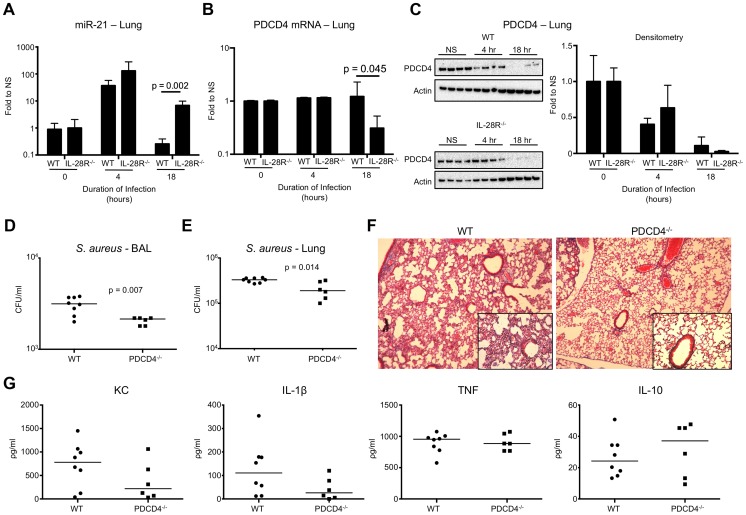
IFNλ regulates PDCD4 in vivo. (A) qRT-PCR analysis of miR-21 in the lungs of WT and IL-28R^−/−^ mice following infection with USA300, µ ± SD. (B) qRT-PCR analysis of PDCD4 mRNA in the lungs of WT and IL-28R^−/−^ mice following infection with USA300, µ ± SD. (C) Western blot analysis of PDCD4 in the lungs of WT and IL-28R^−/−^ mice following infection with USA300, µ ± SD. (D) Numbers (CFU) of USA300 recovered from BAL of WT and PDCD4^−/−^ mice following 18 hours of infection. (E) Numbers (CFU) of USA300 recovered from lung tissue of WT and PDCD4^−/−^ mice following 18 hours of infection. (F) Trichrome stained lungs from WT and PDCD4^−/−^ mice following and 18 hour USA300 infection (original magnification 100x, insert 400x). (G) ELISA analysis of individual cytokines in BAL of WT and PDCD4^−/−^ mice. Data are representative of at least 2 independent experiments.

In vivo, miR-21 expression in WT and IL-28R null mice was not different prior to infection. Expression of miR-21 increased by greater than 10-fold in both WT and IL-28R null mice following four hour infection with USA300 (**Fig. 4A**). By 18 hours following the initial infection miR-21 levels in WT mice returned to uninfected levels, while levels in IL-28R null mice remained significantly higher (p = 0.002). PDCD4 mRNA levels following 18 hour infection inversely correlated with miR-21 expression, and were significantly lower in IL-28R null mice compared to WT (p = 0.045) (**Fig. 4B**). PDCD4 protein expression at the 4 hour time point was reduced in both WT and IL-28R^−/−^ mice compared to unstimulated levels, and reduced further at the 18 hour time point (**Fig. 4C**). PDCD4 protein expression tended to be lower at the 18 hour time point in IL-28R null mice than WT, correlating with elevated levels of miR-21. Thus it appears that following the initial pro-inflammatory innate immune response to *S. aureus* in WT mice, IFNλ signaling suppresses expression of miR-21 maintaining PDCD4 levels. Loss of IL-28R resulted in sustained miR-21 levels and a decrease in PDCD4 gene expression by 18 hours of *S. aureus* infection.

To confirm that PDCD4 expression negatively influences clearance of USA300 from the airway we infected WT and PDCD4^−/−^ mice with 10^7^ CFU USA300 for 18 hours. Significantly fewer organisms were recovered from both the airway (p = 0.007) (**Fig. 4D**) and lung tissue (p = 0.014) (**Fig. 4E**) of PDCD4^−/−^ mice compared to WT. Bacterial clearance correlated with reduced lung pathology (**Fig. 4F**) but not immune cell recruitment into the lung (**Fig. S3**). Expression of the inflammatory cytokines KC and IL-1β, not TNF, were reduced and expression of anti-inflammatory IL-10 was increased in PDCD4^−/−^ mice compared to WT. Although differences in cytokines were not statistically significant, the trends were similar to those observed in IL-28R^−/−^ compared to WT suggesting a link between type III IFN and PDCD4.

### Type III IFN promotes Gram negative pneumonia

Due to previous reports demonstrating improved survival of PDCD4^−/−^ mice during LPS challenge, we hypothesized that IL-28R^−/−^ mice would clear the Gram negative pathogen *P. aeruginosa* PAK more rapidly than WT mice [31]. In vitro, *P. aeruginosa* PAK stimulation (MOI 10, 4 hours) of BMDCs induced increased mRNA levels of IFNλ compared to unstimulated cells (**Fig. S1C**). As shown for MRSA in Figure S1A, loss of IL-28R resulted in reduced IFNλ mRNA levels following *P. aeruginosa* stimulation.

**Figure 5:**
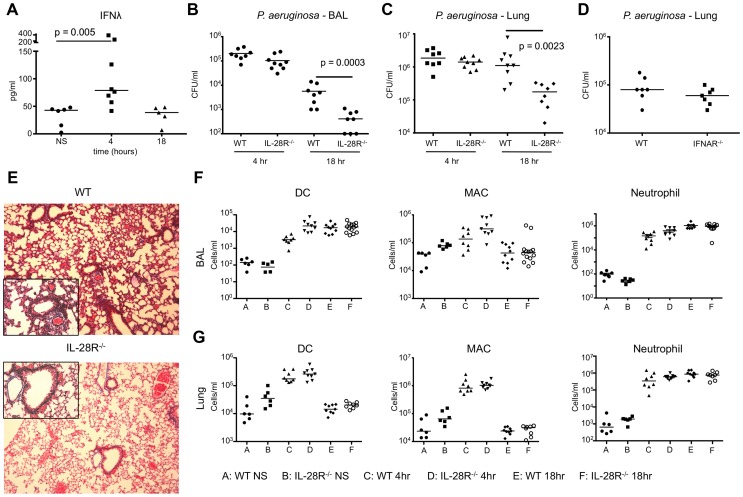
*P. aeruginosa* induction of IFNλ signaling inhibits bacterial clearance. (A) ELISA analysis of IFNλ in BAL of WT mice following 4 and 18 hours of infection with PAK. (B) Numbers (CFU) of PAK recovered from BAL of WT and IL-28R^−/−^ mice following 4 and 18 hours of infection. (C) Numbers (CFU) of PAK recovered from lung tissue of WT and IL-28R^−/−^ mice following 4 and 18 hours of infection. (D) Numbers of PAK recovered from lung tissue of WT and IFNAR^−/−^ mice following 18 hours of infection. (E) Trichrome stained lungs from WT and IL-28R^−/−^ mice following and 18 hour PAK infection (original magnification 100x, insert 400x). (F) Numbers of dendritic cells (DC), macrophages (MAC), and neutrophils in BAL of unstimulated (NS) WT and IL-28R^−/−^ mice or following 4 or 18 hours of infection. (G) Numbers of dendritic cells (DC), macrophages (MAC), and neutrophils in lung tissue of unstimulated (NS) WT and IL-28R^−/−^ mice or following 4 or 18 hours of infection. Data are representative of at least 2 independent experiments.

WT and IL-28R null mice were intranasally infected with 10^7^ CFU PAK and bacterial clearance from the airway and lung tissue monitored at 4 and 18 hours of infection. In vivo, IFNλ mRNA levels (p = 0.0031) (**Fig. S1D**) and protein expression (p = 0.005) (**Fig. 5A**) were significantly increased in WT mice by 4 hours post infection, but were not different than baseline at the 18 hour time point. Significantly fewer bacteria were recovered from BAL (p = 0.0003) (**Fig. 5B**) and lung tissue (p = 0.0023) (**Fig. 5C**) of IL-28R^−/−^ at the 18 hour time point compared to WT, and no differences were observed at the 4 hour time point. Bacterial clearance was not altered in mice lacking the type I IFN receptor IFNAR (**Fig. 5D**). Pathology in the lung was reduced in knockout mice following PAK infection (p = 0.0010) (**Fig. 5E**). Similar to *S. aureus* infection, there were no differences in the numbers of immune cells recruited to the BAL or lung (**Figs. 5F and 5G**).

**Figure 6:**
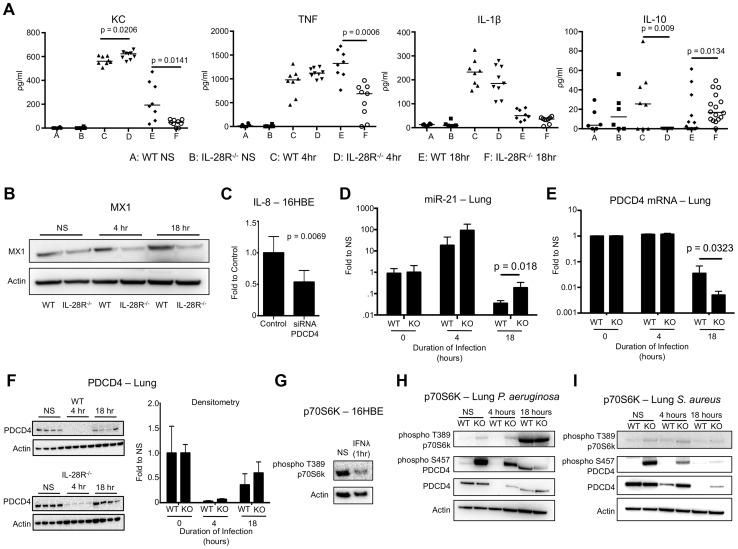
*P. aeruginosa* induced cytokine, miR-21, and PDCD4 expression in WT and IL-28R^−/−^ mice. (A) ELISA analysis of individual cytokines in BAL of WT and IL-28R^−/−^ mice. (B) Western blot analysis of MX1 expression in lungs of WT and IL-28R^−/−^ mice following 4 and 18 hours of infection with PAK. (C) qRT-PCR analysis of IL-8 in 16HBE cells treated with control or PDCD4 siRNA and infected with PAK, µ ± SD. (D) qRT-PCR analysis of miR-21 in the lungs of WT and IL-28R^−/−^ mice following infection with PAK, µ ± SD. (E) qRT-PCR analysis of PDCD4 mRNA in the lungs of WT and IL-28R^−/−^ mice following infection with PAK, µ ± SD. (F) Western blot analysis of PDCD4 in the lungs of WT and IL-28R^−/−^ mice following infection with PAK, µ ± SD. (G) Western blot analysis of phosphorylation of p70S6K in 16HBE cells treated with recombinant IFNλ. (H) Western blot analysis of phosphorylation of p70S6K and PDCD4 in the lungs of WT and IL-28R^−/−^ mice following infection with PAK. (I) Western blot analysis of phosphorylation of p70S6K and PDCD4 in the lungs of WT and IL-28R^−/−^ mice following infection with USA300. Data are representative of at least 2 independent experiments.

BAL fluid was analyzed for the presence of pro- and anti-inflammatory cytokines to determine if IFNλ signaling increased expression of pro-inflammatory cytokines during PAK infection. Significant reductions in expression of pro-inflammatory cytokines KC (p = 0.0141) and TNF (p = 0.0006) were observed in BAL of IL-28R null mice at 18 hours compared to WT (**Fig. 6A**). The anti-inflammatory IL-10 was significantly increased (p = 0.0134) in the IL-28R^−/−^ mice as compared to controls at this time point. No differences in IL-1β production were observed. MX1 levels were lower in IL-28R null mice at 18 hours compared to WT (**Fig. 6B**).

We then determined if the reduced pro-inflammatory cytokine expression in IL-28R^−/−^ mice infected with PAK was also due to alterations in miR-21 and PDCD4. In vitro, PDCD4 siRNA significantly reduced IL-8 expression (p = 0.0069) in 16HBE in response to PAK (**Fig. 6C**). MiR-21 expression in lung tissue of IL-28R null mice was increased (p = 0.0122) compared to WT at 18 hours, demonstrating that loss of IFNλ signaling resulted in increased expression of this miRNA during PAK infection (**Fig. 6D**). PDCD4 mRNA levels were significantly decreased in IL-28R null mice compared to WT correlating with the changes in miR-21 level (p = 0.0323) (**Fig. 6E**). PDCD4 protein in the lung was almost undetectable at the 4 hour time point. PDCD4 levels increased slightly by 18 hours although no differences were observed between knockout and WT mice (**Fig. 6F**).

PDCD4 function can be regulated by p70S6K dependent phosphorylation at serine 475, which inhibits its interaction with AP-1 and eukaryotic translation initiation factor 4A (eIF4A) transcription factors and leads to ubiquitin dependent degradation [22], [33], [44]-[46]. In vitro stimulation of 16HBE monolayers with recombinant IFNλ for 1 hour reduced phosphorylation of p70S6K, suggesting that type III IFN inhibits this regulatory pathway (**Fig. 6G**). In vivo we observed phosphorylation of PDCD4 in uninfected IL-28R^−/−^ mice compared to WT control. In both WT and knockout mice phosphorylation of p70S6K increased following 18 hour PAK infection (**Fig. 6H**), not observed during MRSA infection (**Fig. 6I**). Phosphorylation of PDCD4 in PAK infected WT mice also increased following 18 hours, correlating with phosphorylation of p70S6K. These data suggest that modifications to PDCD4, mediated by both miR-21 and p70S6K, regulate its ability to influence cytokine production during *P. aeruginosa* pneumonia.

## Discussion

Much like the other members of the IFN family, IFNλ is required for host defense against viral pathogens and is capable of inhibiting tumor growth [23]–[28], [30], [47], [48]. There is clearly a major role for IFNλ in the successful clearance of the hepatitis C virus consistent with expression of IL-28R on hepatocytes [49], [50]. Type III IFN has also been linked to killing of the intracellular bacterial pathogen *Listeria monocytogenes,* correlating with reduced colonization of the spleen and liver [51]. Fitting with the abundance of IL-28R in the respiratory tract, we found a major role for IFNλ signaling in the pathogenesis of both *S. aureus* and *P. aeruginosa* pneumonia [21], [28].

In contrast to the beneficial effects of IFNλ in the eradication of viral pathogens, our data suggest that IFNλ promotes a prolonged inflammatory cytokine response to bacterial pathogens that detracts from the efficiency of bacterial clearance and promotes pathology. Mice lacking the type III IFN receptor demonstrated significantly improved clearance of *S. aureus* and *P. aeruginosa* coincident with a reduced duration of the host inflammatory response. Particularly in the pathogenesis of pneumonia, the balance between pro and anti-inflammatory signaling is critical. While the mechanism through which the inflammatory milieu affects bacterial clearance is unclear, our data clearly demonstrate that reducing levels of inflammatory cytokines through modulation of IFNλ or inhibition of IL-1R improves clearance of *S. aureus* and *P. aeruginosa* [11]. Once a sufficient number of phagocytes are recruited to the airways to deal with the pathogens, excessive cytokine production, particularly toxic cytokines such as IL-1β, elicits tissue damage and impairs normal host defenses.

A major effector of IFNλ signaling is PDCD4, which appears to be regulated by multiple pathways in response to infection. A confirmed target of miR-21, PDCD4 influences production of pro-inflammatory signaling through its interaction with AP-1, NF-κB, and eIF4A, and has been linked to the inflammatory microenvironment surrounding tumor cells [10], [12], [29], [31], [33], [52]. Similar to previous findings with LPS, *S. aureus* SpA, through its interaction with TNFR1 upregulates PDCD4 in epithelial cells, promoting pro-inflammatory signaling while inhibiting anti-inflammatory cytokine production. It is likely that additional *S. aureus* pathogen associated molecular patterns (PAMPs) such as the lipoproteins that activate TLR2 also participate.

Following ligation of IL-28R by IFNλ, STAT3 suppresses expression of miR-21 resulting in increased expression of PDCD4. MiRNAs like miR-21 comprise a regulatory system which makes subtle changes to mRNA expression levels, including the genes involved in Toll-like receptor signaling, and therefore are capable of having significant affects on innate immunity [7]–[9], [31], [53]. MiR-21, specifically, is induced by NF-κB signaling and has been implicated in the regulation of TLR2 mediated signaling in both skin and lung inflammation [33], [54]–[57]. We similarly observe rapid increases in miR-21 during the initial 4 hour infection with either *S. aureus* or *P. aeruginosa*, and sustained expression of miR-21 in IL-28R^−/−^ mice was associated with reduced levels of inflammatory cytokines in the airway. As predicted by our in vitro studies, in mice expressing IL-28R reduced levels of miR-21 at 18 hours post infection correlated with prolonged PDCD4 mRNA expression. Mice lacking PDCD4 were better able to clear USA300 from their lungs, similar to previous findings demonstrating PDCD4 null mice are resistant to lethal LPS challenge [31]. Therefore mice lacking IL-28R are less susceptible to *S. aureus* and *P. aeruginosa* induced lung damage due in part to reduced PDCD4 and a shortened inflammatory response.

A second pathway acting through mTOR and p70S6K regulates PDCD4 function by phosphorylating PDCD4, inhibiting its interaction with AP-1 and eIF4A and promoting its degradation [17], [19], [22], [29]. Our data suggest that type III IFN has an inhibitory affect on p70S6K and PDCD4 phosphorylation. In vitro IFNλ treatment of epithelial cells reduced p70S6K phosphorylation. In vivo, PDCD4 was strongly phosphorylated in uninfected IL-28R^−/−^ mice while phosphorylation was not observed in uninfected control mice. Rapid turnover of PDCD4 due to phosphorylation dependent degradation, or an inability of PDCD4 to bind eIF4A in IL-18R null mice could further limit its affects on signaling.

Increases in p70S6K phosphorylation during bacterial infection were IL-28R independent and were only observed 18 hours following infection with *P. aeruginosa*. The ability to activate different regulatory pathways such as p70S6K signaling could contribute to the differences in cytokine induction observed during *S. aureus* and *P. aeruginosa* infections. For example levels of TNF and IL-10 were significantly different between WT and IL-28R^−/−^ mice during PAK not USA300 infections, whereas IL-1β was significantly different during a *S. aureus* infection. Thus IFNλ and PDCD4 are conserved elements in the response to airway pathogens which clearly have other differential effects in inducing innate immunity.

The selective expression of IL-28R, substantially more abundant on murine and human epithelial cells than on immune cells as previously shown and demonstrated by the inability of macrophages to respond to IFNλ (**Fig. S4**), may also be important in the specific cytokine responses evoked by the different pathogens [28]. Epithelial cells are not the primary producers of IL-1β in response to PAK, therefore IL-1β levels in the airways of *P. aeruginosa* infected WT and IL-28R^−/−^ mice were not different [11], [58]. It is unclear which cells in the lung produce IL-1β in response to *S. aureus*. Our data suggest that IL-1β producing cells express IL-28R, as cytokine levels were significant reduced in IL-28R null mice. While we do not propose that IL-1β is the sole cytokine responsible for lung damage in response to *S. aureus*, inhibition of this signaling pathway in WT mice does improve clearance of *S. aureus* and limits the extent of tissue damage. We conclude that while the specific cytokines modulated by type III IFN are different in the context of specific bacterial infections, the overall affect of inhibiting IL-28R dependent signaling is a reduction of lung inflammation and improved bacterial clearance.

The type III IFN pathway is recognized as an important host defense pathway for the eradication of viral infection and tumor growth and the distribution of the receptor at mucosal sites makes it an active participant in the innate immune response to bacterial pathogens as well. While differences in the expression of PAMPs and their receptors, mechanisms of p70S6K and PDCD4 phosphorylation, and ultimately levels of cytokine expression are evident in the models of *S. aureus* and *P. aeruginosa* pneumonia, IFNλ functions as a central regulator of airway inflammation in both types of bacterial pneumonia. Given the pathology associated with excess cytokine induction in the airways, targeting specific components of the type III IFN/PDCD4 regulatory pathway could be a potential therapy for limiting lung damage in the setting of acute airway infection.

## Materials and Methods

### Ethics statement

Animal work in this study was carried out in strict accordance with the recommendations in the Guide for the Care and Use of Laboratory Animals of the National Institutes of Health, the Animal Welfare Act and US federal law. The protocol was approved by the Institutional Animal Care and Use Committee (IACUC) of Columbia University (protocol number AAAC3059).

### Bacterial strains

*S. aureus* strain LAC USA300 and *P. aeruginosa* strain PAK were grown on Luria-Bertani agar at 37°C. For infection, LB broth was inoculated with single colonies and grown overnight at 37°C, diluted 1∶100 in the morning and grown to OD 1.000 (*S. aureus*) or 0.500 (PAK). *S. aureus* cultures grown to OD 1.000 were incubated at 55°C to inactivate the bacteria for heat killed experiments.

### Models of infection

Seven week old C57BL/6 WT, *­*IL-28R^−/−^, IFNAR^−/−^, and PDCD4^−/−^ mice were intranasally inoculated with 50 µl *S. aureus* or *P. aeruginosa* (1*10^7^ CFU/mouse) as previously described [2], [14]. WT and IFNAR^−/−^ mice were previously described, IL-28R^−/−^ mice were provided by Bristol Myers Squibb, and PDCD4^−/−^ mice were from Jackson Laboratories [13]. Control mice received 50 µl of PBS. To determine the affect of IFNλ on cytokine production, mice were intranasally treated with 1 µg recombinant mIL-28 (PBL Interferon Source) in 50 µl PBS or PBS control. The role of IL-1β was determined in mice pretreated i.p. with Anakinra (10 mg/kg, Amgen) for four days and were infected on the fourth day as described previously [59]. Control mice were treated with PBS also given i.p. BAL fluid was harvested 18 hours after infection and used to quantify immune cell populations, cytokine expression, and bacteria CFU. Histology was performed by Columbia University Molecular Pathology core on tissues fixed in 4% paraformaldehyde.

### Cell culture

Bone marrow- derived dendritic cells (BMDCs) were cultured from wild-type and knockout mice as described previously [3]. Human airway epithelial cells (16HBE) were grown as previously described [60]. Human monocytic THP-1 cells were grown in RPMI 1640 medium with 10% fetal bovine serum with penicillin (100 units/ml) and streptomycin (100 µg/ml) and treated with 100 nM phorbol 12-myristate 13-acetate (PMA) to induce terminal differentiation for IFN stimulation studies. On-target control or PDCD4 siRNA (Thermo Scientific) was transfected into 16HBE monolayers using Fugene (Promega) according to the manufacturers instructions. Cells were stimulated with 5 µM *S. aureus* protein A (Calbiochem) or 0.1 µg/ml human recombinant IL-28 or IFNβ (PBL Interferon). In select experiments antibody to the extracellular domain of TNFR1 or control IgG (4 µg/ml) (Santa Cruz Biotechnology Inc.) or STAT3 inhibitor V (50 µM) (Calbiochem) or DMSO control were applied to 16HBEs 1 hour prior to SpA.

### FACS analysis

Enumeration of neutrophil (MHCII^−^Ly6^+^), macrophages (CD11c^+^MHCII^low^), and DC (CD11c^+^MHCII^+^) populations was performed as previously described [1].

### ELISA and immunoblotting

BAL was analyzed for cytokine and chemokine content by ELISA (R&Dbiosystems, PBL Interferon Source, or eBioscience) according to the manufacture’s instructions. Anti-phospho-p70S6K, anti-phospho-PDCD4, anti-PDCD4 (Rockland Immunochemicals), anti-MX1 (Santa Cruz Biotechnology Inc.) and anti-β-actin (Sigma) antibodies followed by secondary antibodies conjugated to horseradish peroxidase (Santa Cruz Biotechnology Inc.) were used to measure expression in human epithelial cells and mouse lung. Protein separation, transfer and immunnoblotting were performed as described [13]. Densitometry was done using Image J (NIH).

### mRNA and miRNA analysis

Total RNA was isolated using *mir*Vana miRNA Isolation Kit (Life Technologies) according to the manufacturers instructions. For mRNA analysis cDNA was generated using the High Capacity cDNA reverse Transcriptase Kit (Applied Biosystems). Quantitative real-time RT-PCR (QRT-PCR) was performed using Power SYBR Green PCR Master Mix in a Step One Plus Thermal Cycler (Applied Biosystems). Mouse primers for PDCD4 were 5′ – ATGGATATAGAAAATGAGCAGAC -3′ and 5′ – CCAGATCTGGACCGCCTATC -3′ and actin were 5′ – CCTTTGAAAAGAAATTTGTCC – 3′ and 5′ – AGAAACCAGAACTGAAACTGG – 3′. Human primers for IL-8 were 5′-TACTCCAAACCTTTCCAACCC-3′ and 5′- AACTTCTCCACAACCCTCTG-3′ and actin were 5′- GTGGGCCGCTCTAGGCACCA-3′ and 5′-CGGTTGGCCTTAGGGTTCAGGGGGG- 3′. IL-8 and PDCD4 expression was normalized to actin. For miRNA analysis cDNA was generated and qRT-PCR was run using NCode miRNA First-Strand cDNA Synthesis and qRT-PCR Kit (Life Technologies) according to the manufacturers instructions. A universal reverse primer for small RNAs was supplied with the NCode kit. The forward primer for miR-21 was 5′-TAGCTTATCAGACTGATGTTGA-3′ and ΔCt values were normalized to the small non-coding RNA U6 5′-GGGCAGGAAGAGGGCCTAT-3′.

### Statistics

Significance of data was determined using a nonparametric Mann-Whitney test. For experiments with greater than one comparison we used a nonparametric Kruskal-Wallis test followed by post-hoc Dunn’s test to correct for multiple comparisons. Statistics were performed with GraphPad Prism software with significance defined as p<0.05. Cytokine, bacterial counts, and immune cell number data are presented as individual points with a bar representing the median value.

## Supporting Information

Figure S1 Induction of IFNλ in bone marrow derived dendritic cells (BMDCs) or Lung tissue. (A) mRNA analysis of IFNλ stimulation following 4 hours of USA300 infection in WT or IL-28R^−/−^ BMDCs, normalized to unstimulated cells (NS) µ ± sd. (B) mRNA analysis of IFNλ stimulation in the lungs of WT mice following 4 or 18 hours of USA300 infection. (C) mRNA analysis of IFNλ stimulation following 4 hours of PAK infection in WT or IL-28R^−/−^ BMDCs, normalized to unstimulated cells (NS), µ ± sd. (D) mRNA analysis of IFNλ stimulation in the lungs of WT mice following 4 or 18 hours of PAK infection. Data are representative of at least 2 independent experiments.
(TIF)

Figure S2 Induction of cytokines by rIL-28. ELISA analysis of cytokines in BAL of wt mice 18 hours following intranasal instillation of rIL-28 (1 µg/mouse). Data are representative of at least 2 independent experiments.
(TIF)

Figure S3 Immune cell populations in WT and PDCD4^−/−^ mice. FACs analysis of dendritic cell, macrophage, and neutrophil populations in the BAL and lung tissue of WT and PDCD4^−/−^ mice following an 18 hour infection with USA300. Data are representative of 2 independent experiments.
(TIF)

Figure S4 Response of human macrophages to type I and III IFN. Western blot analysis of STAT3 phosphorylation in THP-1 cells stimulated with type I (IFNβ) or type III (IFNλ) for 30 minutes or 1 hour. Data are representative of 2 independent experiments.
(TIF)
