# Supplementary figures and images for: Bacterial Pathogens Activate a Common Inflammatory Pathway through IFNλ Regulation of PDCD4
Source: PLoS Pathog. 2013 Oct 3;9(10):e1003682. doi: 10.1371/journal.ppat.1003682 (PMC3789769; doi:10.1371/journal.ppat.1003682)

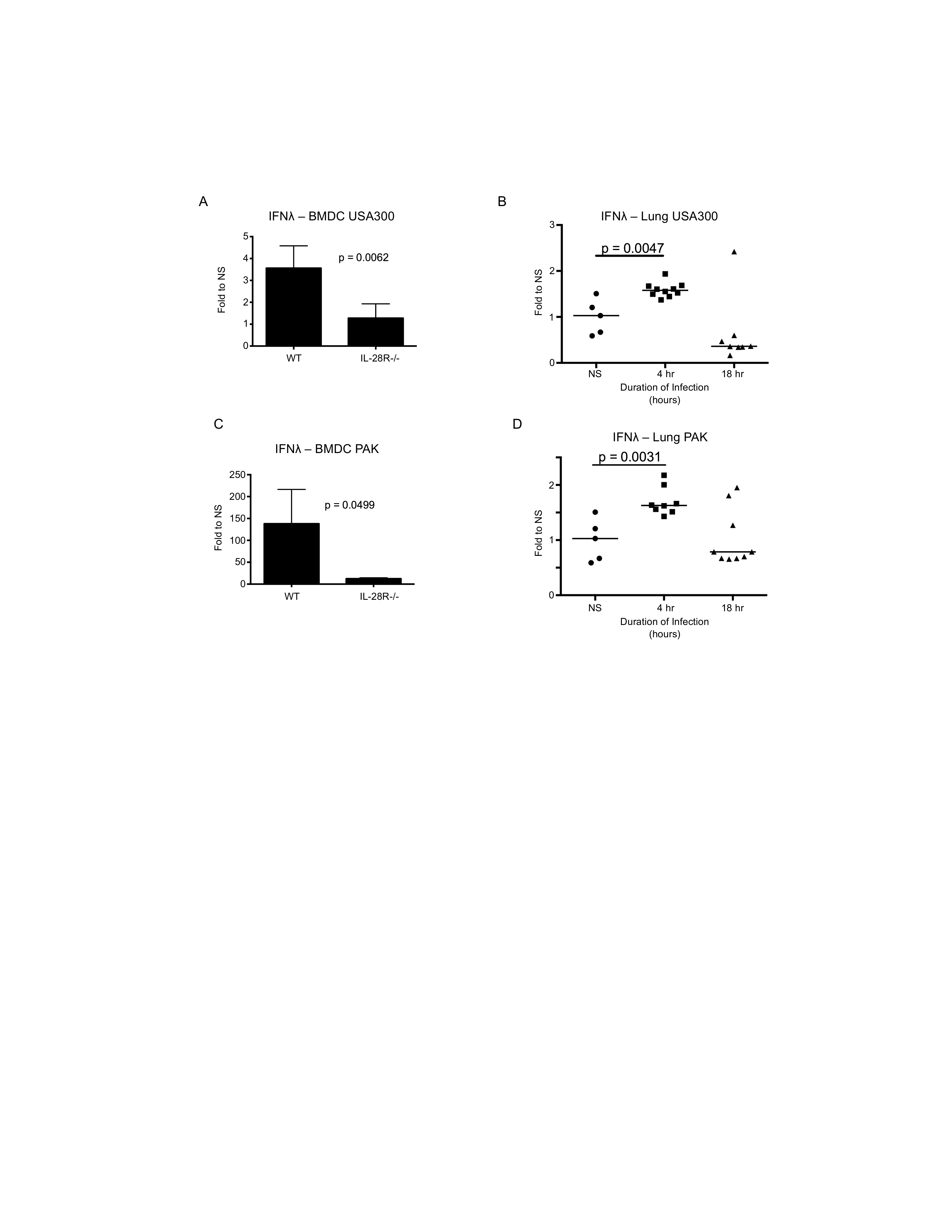

Supplement: Figure S1 — Induction of IFNλ in bone marrow derived dendritic cells (BMDCs) or Lung tissue. (A) mRNA analysis of IFNλ stimulation following 4 hours of USA300 infection in WT or IL-28R−/− BMDCs, normalized to unstimulated cells (NS) µ ± sd. (B) mRNA analysis of IFNλ stimulation in the lungs of WT mice following 4 or 18 hours of USA300 infection. (C) mRNA analysis of IFNλ stimulation following 4 hours of PAK infection in WT or IL-28R−/− BMDCs, normalized to unstimulated cells (NS), µ ± sd. (D) mRNA analysis of IFNλ stimulation in the lungs of WT mice following 4 or 18 hours of PAK infection. Data are representative of at least 2 independent experiments. (TIF) [file ppat.1003682.s001.tif]

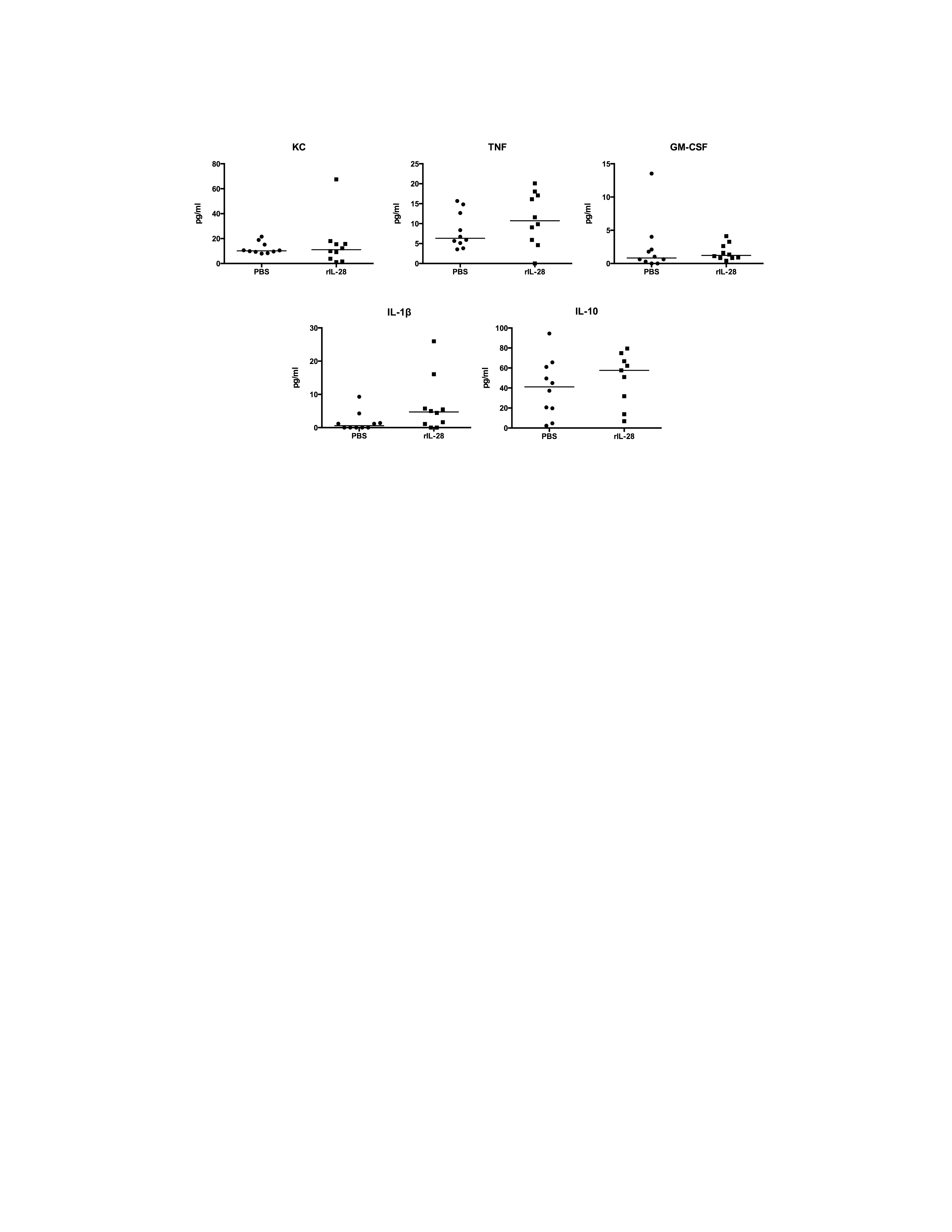

Supplement: Figure S2 — Induction of cytokines by rIL-28. ELISA analysis of cytokines in BAL of wt mice 18 hours following intranasal instillation of rIL-28 (1 µg/mouse). Data are representative of at least 2 independent experiments. (TIF) [file ppat.1003682.s002.tif]

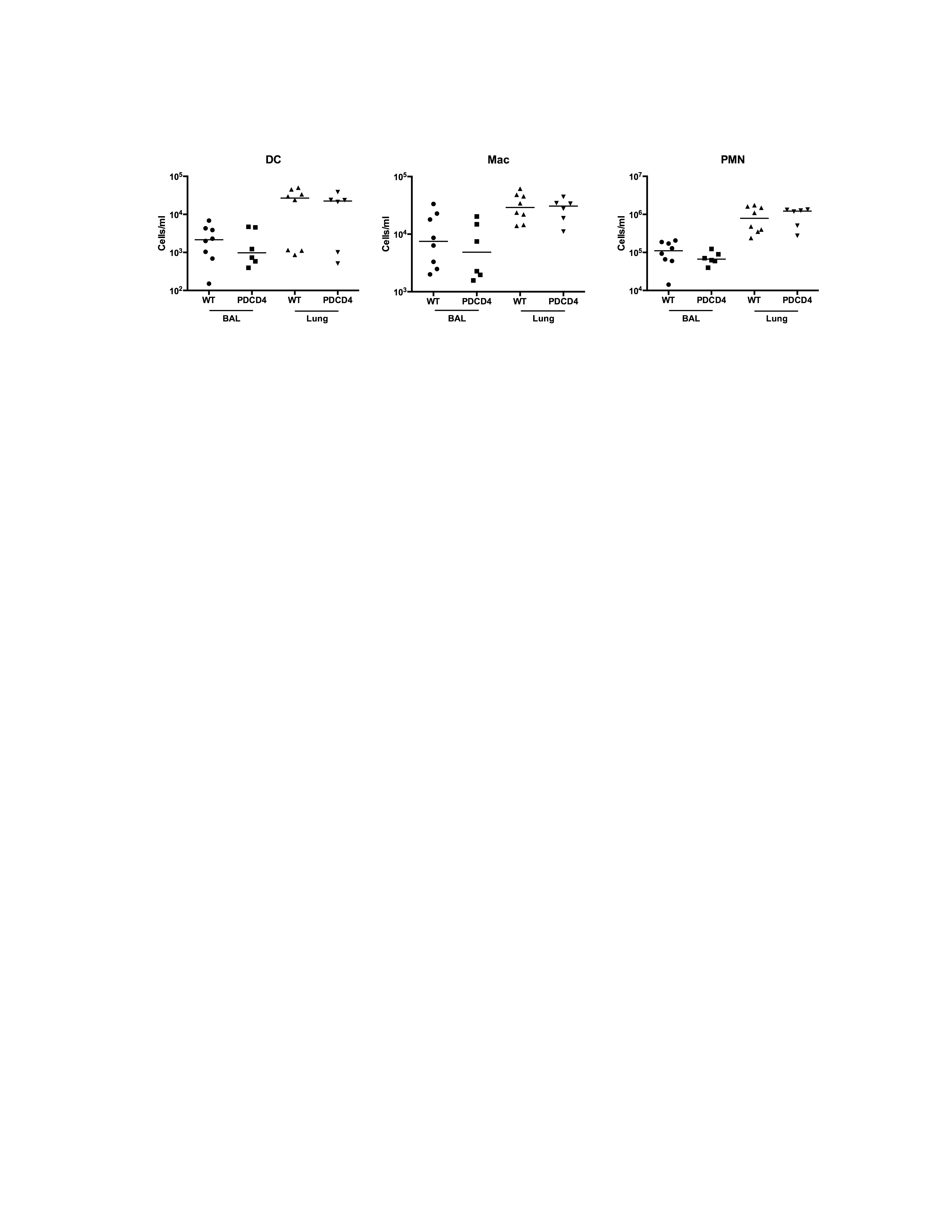

Supplement: Figure S3 — Immune cell populations in WT and PDCD4−/− mice. FACs analysis of dendritic cell, macrophage, and neutrophil populations in the BAL and lung tissue of WT and PDCD4−/− mice following an 18 hour infection with USA300. Data are representative of 2 independent experiments. (TIF) [file ppat.1003682.s003.tif]

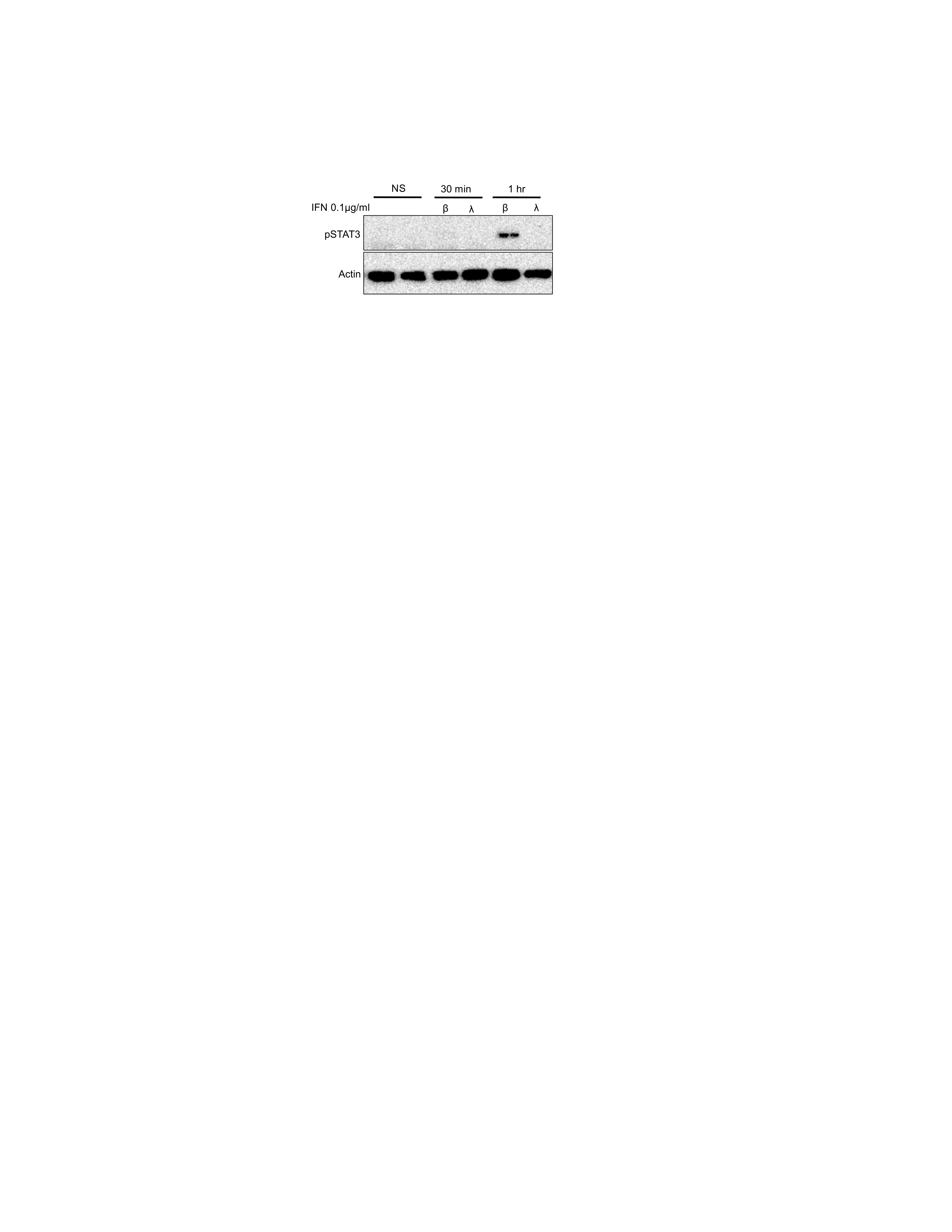

Supplement: Figure S4 — Response of human macrophages to type I and III IFN. Western blot analysis of STAT3 phosphorylation in THP-1 cells stimulated with type I (IFNβ) or type III (IFNλ) for 30 minutes or 1 hour. Data are representative of 2 independent experiments. (TIF) [file ppat.1003682.s004.tif]
